# PyFMLab: Open-source software for atomic force microscopy microrheology data analysis

**DOI:** 10.12688/openreseurope.16550.1

**Published:** 2023-10-30

**Authors:** Javier López-Alonso, Mar Eroles, Sébastien Janel, Massimiliano Berardi, Jean-Luc Pellequer, Vincent Dupres, Frank Lafont, Felix Rico

**Affiliations:** 1 Universite de Lille, CNRS, INSERM, CHU Lille, Institut Pasteur de Lille, U1019-UMR9017, CILL—Center of Infection and Immunity of Lille, Lille, F-59000, France; 2 Aix-Marseille Univ., CNRS, INSERM, LAI, Turing Centre for Living Systems, Marseille, 13009, France; 3 LaserLab, Department of Physics and Astronomy, Vrije Universiteit Amsterdam, Amsterdam, 1081HV, The Netherlands; 4 Optics 11 B.V, Amsterdam, 1101BM, The Netherlands; 5 Univ. Grenoble Alpes, CEA, CNRS, IBS, Grenoble, F-38000, France

**Keywords:** force spectroscopy, Elasticity, Viscoelasticity, Young’s modulus, Biological Samples, cell mechanics, tissue mechanics, soft matter

## Abstract

**Background:**

Atomic force microscopy (AFM) is one of the main techniques used to characterize the mechanical properties of soft biological samples and biomaterials at the nanoscale. Despite efforts made by the AFM community to promote open-source data analysis tools, standardization continues to be a significant concern in a field that requires common analysis procedures. AFM-based mechanical measurements involve applying a controlled force to the sample and measure the resulting deformation in the so-called force-distance curves. These may include simple approach and retract or oscillatory cycles at various frequencies (microrheology). To extract quantitative parameters, such as the elastic modulus, from these measurements, AFM measurements are processed using data analysis software. Although open tools exist and allow obtaining the mechanical properties of the sample, most of them only include standard elastic models and do not allow the processing of microrheology data. In this work, we have developed an open-source software package (called PyFMLab, as of python force microscopy laboratory) capable of determining the viscoelastic properties of samples from both conventional force-distance curves and microrheology measurements.

**Methods:**

PyFMLab has been written in Python, which provides an accessible syntax and sufficient computational efficiency. The software features were divided into separate, self-contained libraries to enhance code organization and modularity and to improve readability, maintainability, testability, and reusability. To validate PyFMLab, two AFM datasets, one composed of simple force curves and another including oscillatory measurements, were collected on HeLa cells.

**Results:**

The viscoelastic parameters obtained on the two datasets analysed using PyFMLab were validated against data processing proprietary software and against validated MATLAB routines developed before obtaining equivalent results.

**Conclusions:**

Its open-source nature and versatility makes PyFMLab an open-source solution that paves the way for standardized viscoelastic characterization of biological samples from both force-distance curves and microrheology measurements.

## Plain language summary

Just like we can test the ripeness of fruit by touching it, we can use our hands to gently touch an object and determine if it is soft or stiff. Doctors use this technique, called palpation, to explore our organs and check for signs of disease. We can think about doing something similar, but on a much smaller scale — the nanoscale — so small that you cannot even see it with your naked eye. Atomic force microscopy (AFM) allows to apply palpation at the nanoscale.

AFM is a powerful tool that allows scientists to examine incredibly small objects, like individual cells or molecules. AFM uses super sensitive “fingers” to touch and explore things that are too small to be seen under a regular microscope. During the European project Phys2BioMed, we explored how to apply AFM to diagnose diseases using nanopalpation. For example, touching samples from biopsies of patients and determining how soft or stiff they are.

Here's the catch: there is not a single, standardized method or software that can efficiently process all the data obtained from AFM. It's a bit like having a lot of different languages but no universal translator. Just like a scale or a measuring cup are standardized, scientists need to analyze AFM data accurately and consistently. This is crucial to ensure reliable comparisons between results obtained by different researchers on different instruments, something particularly important when the results are to be used for diagnostic or predictive purposes.

To help tackle this problem, we have developed PyFMLab. This software is a reliable and easy-to-use tool that translates AFM data into insights about the tiny structures being studied. By providing a standardized, open-source, modular and accessible way to analyze AFM data, PyFMLab democratizes access to this field of Biophysics, paving the way for clinical applications of AFM.

## Introduction

In recent years, the study of cellular and extracellular matrix (ECM) mechanics has increased considerably due to the realization of its fundamental role in several physiological and pathological processes like cell division, migration, differentiation and malignancy ( Engler *et al.*, 2006; Rianna *et al.*, 2020). Amongst other techniques, atomic force microscopy (AFM) has proven to be a powerful tool to characterize the mechanical properties of living cells and ECM ( Lekka *et al.*, 2023a; Lekka *et al.*, 2023b) and it is considered as a standard tool in the field. Despite its popularity, standardization of sample preparation, measurement and data analysis protocols are still lacking.

To overcome some of these limitations, several efforts have been initiated towards AFM probe calibration procedures ( Sader *et al.*, 2016; Schillers *et al.*, 2017), and open source or free AFM data analysis tools like Gwyddion ( Necas & Klapetek, 2012), TopoStats ( Beton *et al.*, 2021), AtomicJ ( Hermanowicz *et al.*, 2014), PyJibe ( Müller *et al.*, 2019), ViscoIndent ( Efremov *et al.*, 2020) and Rheos ( Kaplan *et al.*, 2019).

The analysis of AFM force measurements is generally carried out using either home-made ( Carl & Schillers, 2008; Chen *et al.*, 2022; Domke & Radmacher, 1998; Kontomaris *et al.*, 2023; Lekka *et al.*, 1999; ) or instrument-associated commercial software. Although these tools allow to extract the topographical and mechanical properties of the sample, most of these packages are either not open source, limited to the most standard models, not compatible with other manufacturers data formats, do not allow obtaining viscoelastic parameters or do not support microrheology data.

The most common approach to determine the elasticity of soft biological samples is the acquisition of AFM force-distance curves (FDCs), to then fit an elastic contact model, usually to the approach segment of the curve, and obtain the apparent Young’s modulus (E). For determining the viscoelasticity of the sample, two main approaches are possible:

• FDC (time-domain method): where a viscoelastic model is fitted to the full FDC (approach and retract) ( Brückner *et al.*, 2017; Efremov *et al.*, 2020; Sanchez *et al.*, 2021) or to certain segments (approach, pause, steps ( Yango *et al.*, 2016)).
• Microrheology (frequency-domain method): consisting in the force response analysis of small amplitude z oscillations at different frequencies to determine the complex shear modulus ( Alcaraz *et al.*, 2003).

Most available AFM open-source and commercial software tools allow to compute E from FDCs but not viscoelastic parameters. Amongst open-source tools, only VisconIndent natively supports rheology data, but even that is limited to time domain analysis, which requires a priori knowledge regarding the constitutive behavior of the sample under test. A turn-key solution that encompasses both time and frequency domain viscoelastic analysis, as well as microrheology related signal correction procedures is still missing. Making this type of analysis more accessible is important, as recent works have shown the time dependent response of ECM plays a crucial role in tissue organization ( Elosegui-Artola *et al.*, 2023). PyFMLab has been developed with these needs in mind.

## Software architecture

**Figure 1.**
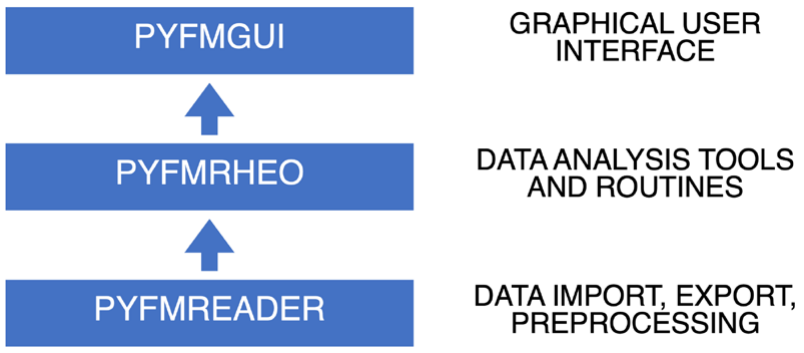
Diagram representing how the libraries composing PyFMLab are organized.

The software functionalities were split into different self-contained libraries to help organize the code while making it more readable, maintainable, testable and reusable. Similarly to the AFM data analysis PyJibe developed by Paul Muller ( Müller *et al.*, 2019), the functionalities of PyFMLab are split on three main Python libraries organized in a hierarchical manner ( Figure 1):

### PyFMReader

 The source code for PyFMReader is available (from zenodo López-Alonso, 2023b) (also available from GitHub)

This library includes methods to load and pre-process data from different AFM manufacturer files, making FDCs, topography data and metadata accessible to the user. At the core of this library, is the universal file format (UFF) object (manuscript in preparation). The files from different instruments are parsed and the metadata, force curve and image data are loaded into the UFF object.

PyFMReader can be used as a stand-alone library to load data acquired by Bruker® instruments running JPK SPM software v4.2 – v7.0 (.jpk-force, .jpk-force-map, .jpk-qi-data) or Nanoscope software v7.0 – v9.0 (.spm, .pfc). PyFMReader allows exporting to UFF txt data files. Interactive Jupyter notebooks are provided with examples on how to load files and access loaded data.

**Figure 2.**
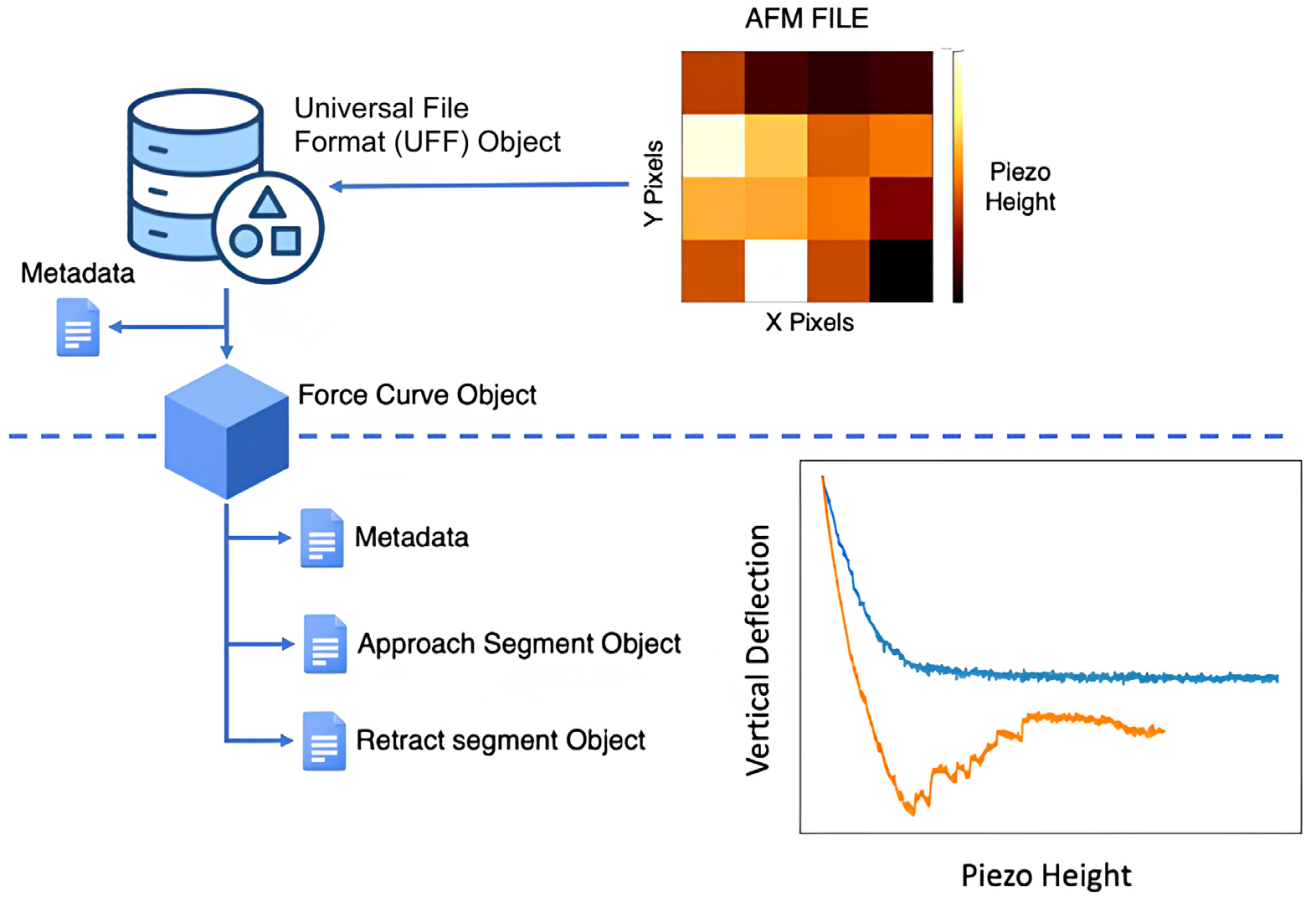
Hierarchical data organization used in PyFMLab. UFF stands for universal file format.

To allow flexibility in implementing functionalities, each file (map or single force curve) is stored within a UFF object. This implementation results in a hierarchical data organization ( Figure 2) where UFF objects point to the different force curve objects and each force curve object points to distinct segment objects (approach, pause, modulation and retract segments). The name of the file is used as the id to relate all data objects from each file.

### PyFMRheo

The source code for PyFMRheo is available (from zenodo López-Alonso, 2023b) ( also available from GitHub)

As the main building block of PyFMLab, this library includes a wide variety of models and predefined routines for the nanomechanical characterization of biological samples, correction for hydrodynamic drag, calibration of the z-piezo for oscillatory measurements and non-contact calibration of AFM probes. As PyFMReader and PyFMRheo have been developed with the intention of being used together, the predefined routines available in PyFMRheo expect input FDCs and thermals as objects defined by PyFMReader. Nevertheless, this library can be used on its own, with the user being able to decide which library to use to load their data, which allows it to be used for the analysis of any data acquired on lab made or other commercial instruments. All models and tools required for processing AFM force curves and thermal tunes are fully accessible, allowing the users to compose their own custom data processing scripts. Examples on how to use each of the predefined routines are provided as interactive Jupyter notebooks.

***Elastic models.*** Contact models describing the force ( *F*) versus indentation ( *δ*) relationship allowing to obtain the Young’s modulus ( *E*) from the approach segment of FDC for several AFM tip geometries have been included (see chapter 2.2 Contact Mechanics Lacaria *et al.* in Lekka *et al.,* 2023a):

Additionally, the library includes a model considering the finite thickness of the sample ( Table 2) developed by Garcia and Garcia ( Garcia & Garcia, 2018), where the thickness of the sample is considered when determining the Young’s modulus.

Due to the complexity of automatically determining the thickness of the sample at each pixel, in particular for force maps, the models incorporating Garcia and Garcia bottom effect correction are implemented in the PyFMRheo analysis library but are not accessible through the graphical user interface (GUI).

*Default routine implementation*

1. The raw data of the curve is pre-processed to obtain the tip position ( *δ*) by subtracting the cantilever vertical deflection ( *d*) to the piezo position ( *z*).
2. An estimate of the point of contact (PoC) is computed using the ratio of variances (RoV) method based on Gavara, 2016. This is computed in two small windows before and after each point of the deflection signal ( *d*):
$$RoV_{i}\;=\;\frac{var\left(d_{i+1}:d_{i+N}\right)}{var\left(d_{i–N}:d_{i–1}\right)},$$
  Where *RoV _i_* is the ratio of variances at each data point *i*, *var* is the variance, *d* is the deflection signal and *N* is the window size inputted by the user.
3. The spring constant and initial estimation of the PoC are used to obtain the force *vs* indentation curve.
4. Using the parameters defined by the user, the corresponding model is fitted to the force *vs* indentation curve to obtain the refined PoC and the Young’s modulus.

***Viscoelastic models***. Several models have been proposed to determine the viscoelastic force-indentation relationship of a sample. These models are numerical ( Efremov *et al.*, 2017) or analytical ( Brückner *et al.*, 2017; Garcia *et al.*, 2020; Eroles *et al.*, 2023) solutions and are based on the seminal works by Lee and Radock ( Lee & Radok, 1960), Graham ( Graham, 1967) and Ting ( Ting, 1966; Ting, 1968). The selection and implementation of the models in PyFMLab were performed focusing on the determination of the mechanical properties of living mammalian cells and extracellular matrices. Such samples have been shown to exhibit a power law response both at low and high frequencies ( Balland *et al.*, 2006; Fabry *et al.*, 2001; Jorba *et al.*, 2017; Jorba *et al.*, 2019; Rigato *et al.*, 2017). PyFMRheo includes both numerical ( Table 3) and analytical ( Table 4) models for a power law response material.

In the models, *F* is the force acting on the cantilever tip, *δ* is the indentation which is a function of the time, *t*. Following (Brückner *et al*.), the time *t*_1_ is obtained from the auxiliary function determined by:

$$\int_{t_{1}\left(t\right)}^{t}E\left(t–\xi \right)\;\frac{\partial \delta }{\partial \xi }\;d\xi \;=\;0,$$

where *ξ* is the dummy time variable required for the integration, *E*(t) is the Young’s or elastic relaxation modulus described by a power-law rheology (PLR) model:

$$E\left(t\right)\;=\;E_{0}{\left(\frac{t}{t_{0}}\right)}^{–\beta },$$

where *E*_0_ is the scaling factor or instantaneous elastic modulus, *t*_0_ an arbitrary time scale assumed to be 1s, and *β* is the power-law exponent or fluidity ( *β* = 0 for material with solid-like behaviour, *β* = 1 for material with fluid-like behaviour).

Assuming that the indentation is proportional to the different approach and retract velocities, *t*_1_ is found to be:


$$t_{1}\;=\;t\;–\;{\left(1+\frac{v_{r}}{v_{a}}\right)}^{\frac{1}{1\;–\beta }}\left(t\;–\;t_{m}\right),$$

where *v _a_* is the constant approach velocity, *v _r_* is the constant retract velocity ( Brückner *et al.*, 2017).

*C~* is the geometry dependent coefficient. The definition of this parameter based on the tip geometry is included in Table 5.

*Default viscoelastic routine implementation*

1. The raw data of the curve is pre-processed to obtain the tip position by subtracting the cantilever vertical deflection to the piezo position.
2. An estimate of the point of contact (PoC) is computed using the ratio of variances (RoV) method based on Gavara, 2016.
3. The spring constant and initial PoC estimation are used to obtain the force vs. indentation curve.
4. The elastic model corresponding to the AFM tip geometry is fitted to the approach segment of the force vs. indentation curve to obtain an estimation of the Young’s modulus and a better estimation of the PoC.
5. The corresponding viscoelastic model is then fitted to the full approach-retract cycle of the force vs indentation curve to obtain the power-law fluidity exponent ( *β*), the instantaneous elastic modulus ( *E*_0_) and the PoC. The viscous drag force (force felt by the cantilever due to the viscosity of the medium) can be corrected, from the correction factor specified by the user and the respective approach and retract velocities.

***Determination of the complex shear modulus from active microrheology measurements***. Dynamic mechanical analysis (DMA), or microrheology measurements, consist of applying deformations in a cyclical manner to a material in the following manner:

1. The cantilever approaches and indents the sample.
2. The cantilever oscillates at a single frequency. This step is repeated in order to measure all the desired frequencies.
3. The cantilever is retracted from the sample.

These cyclic forces result in variable deformations. As the stress (force applied to an area of the material) or strain (material deformation) applied is oscillatory (sinusoidal), the response of the sample is also sinusoidal. If a material is purely elastic, the strain and stress will be perfectly synchronous, at all times, meaning that, when the force is applied, the material will deform, and when the force stops being applied, the sample will recover its original shape. If the material is purely viscous, an applied stress will induce a strain response that is both delayed in time and not recovered once the application of stress ceases. Generally, a material will exhibit both elastic and viscous behaviors, resulting in a strain response that will lag (phase angle 0 – 90°) behind the applied stress. From DMA, the following frequency dependent parameters can be obtained:

• Complex modulus ( G*): Overall resistance of the material to deformation including a real part (elastic, *G′*) and an imaginary part (viscous, *G″*).
• Storage modulus ( *G*′): Elasticity of the materials and its ability to store energy.
• Loss modulus ( *G*): Viscosity of the material and its ability to dissipate energy.
• Loss tangent ( *η*): Degree of solid- or liquid-like mechanical behavior of the material.

Table 6 shows the values for *G*′, *G*″ and *η* for purely elastic and purely viscous materials. Mammalian cells and extracellular matrices (ECM) are viscoelastic materials, with both an elastic and viscous component. In 2003 Alcaraz *et al.* defined a methodology for the microrheological characterization of biological samples with AFM. When a sample is cyclically indented in AFM, a certain stress is applied to the sample, causing it to deform. Both the stress and the deformation of the sample can be measured through force-indentation curves, where the force can be used to monitor the stress applied, and the indentation signal can be used to monitor the corresponding deformation of the sample.

Models for two AFM tip geometries have been implemented and are shown in Table 7.

The default routine expects data which has been acquired by indenting the sample and applying single frequency oscillations at consecutive segments, as shown in Figure 5.

*Default rheology routine implementation*

1. The raw data of the curve is pre-processed to obtain the tip position by subtracting the cantilever vertical deflection to the piezo position.
2. The force curve is pre-processed to obtain the piezo position *vs* tip position.
3. An estimate of the point of contact (PoC) is computed from the approach segment using the ratio of variances (RoV) method based on ( Gavara, 2016). This allows determining the value of the working indentation.
4. If the user provides piezo calibration data and selects the option, the amplitude and phase of the z-piezo signal are corrected.
5. Both signals are then detrended by subtracting the rolling average of the signal with a window size equal to the number of points of each period.
6. The transfer function ( *H _d_*) is computed considering the ratio of the Fourier transforms of the small amplitude indentation ( *δ*( *ω*)) as the input signal and the measured force ( *F*( *ω*)) as the output signal.
$$H_{d}\;=\;\frac{F\left(\omega \right)}{\delta \left(\omega \right)}$$
7. If the user provides a value for the drag force at contact, the transfer function values are corrected accordingly.
8. G*( *ω*) is computed using the corresponding frequency domain rheological contact model. From the values of G*( *ω*), *η*( *ω*) is computed.

***Z-Piezo characterization***. When characterizing the microrheological properties of cells or ECM, any delay between the force and indentation signals inherent to the instrument will result in an over- or underestimation of the viscoelastic properties of the sample. By acquiring oscillatory measurements in a non-deformable substrate, like glass, the inherent phase angle ( *ϕ _PZT_*) between the deflection and z movement signals of the instrument can be measured and used to correct the measurements acquired on the sample.

$$G{*}_{corr}=\;G*e^{–i\phi_{PZT}}$$

In addition to the previous correction, in this routine, the ratio between the vertical deflection and the z-piezo signals is computed. This value can then later be used to correct the z-piezo signal acquired on the sample.

$$z_{corr}=zA_{corr}$$

*Default z-piezo characterization routine implementation*

1. Both the z-piezo displacement ( *z*) and the vertical deflection of the cantilever are detrended ( *d*) by subtracting the rolling average of the signal with a window size equal to the number of points of each period.
2. The transfer function is computed considering *z _PZT_* as the input signal and *d _v_* as the output signal.
$$H_{d}=d/z$$
3. From the transfer function, the phase between *z* and *d* signals at each applied frequency are obtained.
4. Finally, the signal amplitude quotient between *z* and *d* signals is computed.
$$A_{corr}=\frac{A_{d}}{A_{z}}$$

***Determination of viscous drag coefficient***. To minimize the noise in the measurements and to provide a suitable environment when working with living cells, most AFM microrheological measurements are performed in liquid. Therefore, when the cantilever oscillates, the surrounding fluid applies force on the cantilever by adding resistance to the motion (hydrodynamic drag). In the same manner as the piezo lag, the hydrodynamic drag results in an overestimation of the viscous properties of the measured sample. To account for this effect, Alcaraz and collaborators ( Alcaraz *et al.*, 2002) proposed a correction applicable to AFM measurements on soft samples in liquid at low Reynold numbers (Re < 1). They approached this problem by modelling the AFM cantilever as a sphere close to a rigid wall. The characterization of the hydrodynamic on a spherical body close to a rigid wall has been addressed by multiple authors ( Brenner, 1961; Cox & Brenner, 1967). Based on these works, Alcaraz and collaborators estimate the drag force *b*( *h*) when approaching the sample to be:

$$b\left(h\right)=\frac{6\pi \eta a_{eff}^{2}}{\left(h+h_{eff}\right)}$$

Where *η* is the viscosity of the fluid, *a _eff_* is the effective radius of the cantilever, *h _eff_* is the effective height of the tip and *h* is the distance between the tip and the sample.

Using this scaled spherical model, the drag force at contact ( *b*(0)) can be estimated by measuring the drag force *b*( *h*) at different cantilever-sample separations and extrapolating the data to h = 0.

To compute *b*( *h*) the default routine expects data acquired with a specific protocol:

1. The AFM tip approaches and indents the sample (cell, tissue, gel, *etc.*).
2. The cantilever retracts a certain distance defined by the user.
3. The cantilever is oscillated at a single frequency defined by the user.
4. Steps 2 and 3 are repeated until the maximum desired distance from the sample has been reached.

An example of data collected using this protocol is shown on Figure 6.

*Default viscous drag routine implementation*

1. If the user provides piezo calibration data and selects the option to correct the amplitude of the z-piezo, the signal is corrected.
2. The transfer function ( *H _d_*) is computed considering the indentation ( *δ*) as the input signal and the measured force ( *F*) as the output signal.
$$H_{d}\;=\;\frac{F\left(\omega \right)}{\delta \left(\omega \right)}$$
3. If the user provides piezo calibration data, the transfer function values are corrected using the piezo amplitude and lag.
4. *b*( *h*) is then computed using the following expression:
$$b\left(h\right)=\frac{H_{d}}{\left(2\pi \omega \right)}$$

***Non-contact cantilever calibration***. Many indirect spring constant calibration methods have been developed, with the Sader method being the most widely used, thanks to its ease of use and applicability to different cantilever geometries (rectangular, V-shaped) ( Sader *et al.*, 1999; Sader *et al.*, 2005; Sader *et al.*, 2012). This method relies on measuring the quality factor ( *Q*) and resonant frequency ( *ω _R_*) from the thermal spectrum of the cantilever to estimate the spring constant ( *k*) by relying on hydrodynamic theory. When the cantilever oscillates, the surrounding fluid (air, water, *etc.*) applies force on the cantilever in two ways: by adding resistance to the motion (hydrodynamic drag) and by adding mass to the cantilever. By relating the measured *ω _R_* and *Q* factor of the cantilever oscillating in a fluid to the values of the cantilever oscillating in vacuum, an analytical model to determine the spring constant of the cantilever for the first eigenmode (k _1_) can be derived, by taking into consideration the geometry of the cantilever together with the density and viscosity of the fluid ( Sader *et al.*, 2005). The spring constant of a rectangular cantilever can be determined applying the following formula:

$$k=0.1906\rho b^{2}L\Gamma_{i}\left(\omega_{R}\right){\omega_{R}}^{2}Q$$

Where *ρ* is the density of the medium, *b* is the thickness of the cantilever, *L* is the length of the cantilever, *Γ _i_* are the imaginary components of the hydrodynamic function, *ω _R_* is the resonance frequency of the cantilever and *Q* is the quality factor of the cantilever. Although this expression is limited to rectangular cantilevers, the Sader method was later extended to cantilevers of arbitrary shape ( Sader *et al.*, 2012)

With the goal of allowing to calibrate the spring constant of cantilevers with a reference standard, Sader *et al.* developed a virtual instrument (that allows the user to obtain the spring constant of a cantilever regardless of its shape by measuring *ω _R_* and *Q*. This method is referred to as the global calibration initiative (GCI) ( Sader *et al.*, 2012; Sader *et al.*, 2016).

In 2006, Higgins *et al.* proposed a non-contact method to obtain the optical lever sensitivity (or deflection sensitivity) from the thermal spectrum of the cantilever, assuming that the spring constant remains unchanged in air and liquid. This approach requires a previous calibration of the spring constant in air with the Sader or other method ( Higgins *et al.*, 2006). As this method yields a deflection sensitivity different to the one obtained by contact-based methods, the result is then multiplied by a correction factor to obtain the correct value.

Although the Sader and GCI methods enhance the precision of nanomechanical measurements when compared to conventional contact-based approaches ( Sader *et al.*, 2012; Sader *et al.*, 2016), they both assume cantilevers with a high *Q* value, which is not always the case for most cantilevers in liquid. For this reason, in 2020, Sumbul and collaborators ( Sumbul *et al.*, 2020) assessed the accuracy and precision of these methods. In this study, they observed that using the single harmonic oscillator (SHO) model to fit the thermal spectra together with the GCI method was less prone to systematic uncertainties and provided higher accuracy in the determination of *k* and the deflection sensitivity ( Sumbul *et al.*, 2020). The implementation of the calibration routine in this package has been based on these works.

*Default spring constant calibration routine implementation*

1. The SHO model is fit to the first eigenmode peak of the thermal data to obtain the white noise of the signal ( *A _white_*, the amplitude of the peak ( *B*), the resonance frequency ( *f _R_* and *Q* factor of the cantilever.
$$S_{SHO}\left(f\right)=A_{white}^{2}+\frac{B^{2}f_{R}^{4}}{Q^{2}}{\left[{\left(f^{2}–f_{R}^{2}\right)}^{2}+\frac{f^{2}f_{R}^{2}}{Q^{2}}\right]}^{–1}$$
2. From these values the spring constant can be computed using the general Sader method. This step is only valid for rectangular and V-shaped cantilevers.
3. If the user provides thermal data acquired in air, their username and password for the GCI web application, the software will get the GCI spring constant through the application programming interface of the GCI web application.
4. The deflection sensitivity (or inverse of the optical lever sensitivity, invOLS) is computed using the method proposed by Higgins *et al.* and Sumbul *et al.* The user can specify the factors to correct for the static versus dynamic deflection (sometimes referred as *χ*) and the fundamental oscillation mode (sometimes referred as *β*) of the cantilever ( Chighizola *et al.*, 2023). If no correction factor is specified, the default values ( $\sqrt{\beta }/\chi$ ) of 0.90 (rectangular) and 0.87 (V-shaped) are used ( Pirzer & Hugel, 2009; Stark *et al.*, 2001; Sumbul *et al.*, 2020).

### PyFMGUI

The source code for PyFMGUI is available (from zenodo López-Alonso *et al.,* 2023b) ( also available from GitHub)

To make the software more accessible to the end user, a GUI has been developed. This GUI allows the user to load, visualize and process data using the methods available in the PyAFMReader and PyAFMRheo libraries. Multiprocessing has been implemented to speed up loading and processing of large files.

The multiple-document interface (MDI) has been chosen as the model to develop the GUI. This model allows the user to have multiple analysis windows open within the application. The cross-platform Qt5 framework was chosen to program the GUI. As PyFMLab is programmed in Python, the library PyQT5 was used to access the bindings for Qt5. Thanks to its speed, PyQTGraph was used to produce the plots of the application.

As a free, cross-platform, and versatile high-level programming language, Python has gained widespread adoption within the scientific community. This popularity has, in turn, spurred the development of a rich ecosystem of Python scientific packages, enabling tasks such as data visualization, machine learning, natural language processing, and complex data analysis, among others. Table 8 provides a list of the widely recognized and well-supported Python packages used to develop PyFMLab.

For manipulating arrays and matrices, numerical computing, and linear algebra Numpy ( Harris *et al.*, 2020) was the library selected. The Pandas ( McKinney, 2010) library was used to organize the results into a tabular form and export them as coma separated values (.csv) files. The Scipy ( Virtanen *et al.*, 2020) library was used in PyFMLab for signal detrending and the implementation of the gaussian filter, one-dimensional discrete Fast Fourier Transform, Beta function, Gamma function and Besel function. Finally, for Non-Linear Least-squares minimization using the Levenberg-Marquardt algorithm, the Lmfit ( Newville *et al.*, 2023) library was used.

## Software validation

### Cell culture

HeLa cells (ATCC, Manassas, VA, USA) were cultured in Minimum Essential Medium (MEM) (ThermoFischer, Waltham, MA, USA) containing 10% fetal bovine serum, 10% L-Glutamine and 1% Penicillin/Streptomycin. Cells were then seeded in 35 mm diameter glass-bottom Petri dishes (WillCo, Amsterdam, NL) and incubated overnight at 37ºC and 5% CO _2_.

### AFM measurements

For measuring the mechanical properties of cells, maps of force curves, with a size of 30 µm x 30 µm and a resolution of 4 x 4 pixels, were acquired on the nucleus area of 20 cells using a NanoWizard III (Bruker, Santa Barbara, CA, USA). The maps were acquired using a SAA-SPH-5UM probe (hemispherical tip, 5 µm radius, 0.192N/m spring constant) (Bruker, Camarillo, CA, USA) using the following parameters: 1 nN force setpoint, 4 μm ramp size and 20 μm/s ramp speed. During all measurements, indentations ~500 nm were targeted to avoid the bottom effect of the substrate.

We acquired two datasets. The first dataset (A, simple force curves) involved approaching and retracting the tip at a constant velocity to collect data on 20 HeLa cells for analysis using elastic and viscoelastic models. The second dataset (B, oscillatory measurements) involved approaching the tip at a constant velocity, oscillating the tip in contact with the sample and retracting at constant speed and was used to collect data on 20 HeLa cells for analysis using complex shear modulus models. In this latter case, sinusoidal oscillations of 15-nm amplitude at 0.6 Hz, 1 Hz, 10 Hz, 60 Hz, 120 Hz and 200 Hz were applied during the z-piezo characterization and microrheological measurements on cells. To compute the viscous drag coefficient at contact, the cantilever was oscillated at 500 Hz with 15-nm amplitude at tip-sample distances between 500 nm and 3 µm from the contact surface.

### AFM data processing

Data was processed using three software packages:

• PyFMLab: The software package developed in this work.
• MATLAB (RRID: SCR_001622) Routines: Custom routines developed in MATLAB and applied before for elastic ( Rico *et al.*, 2005) and viscoelastic ( Gerum *et al.*, 2022) FDCs data processing.
• JPK DP (v.7.1.23): Commercial software developed and provided by Bruker, used to visualize and process data acquired with the NanoWizard AFM platform.

**Figure 3.**
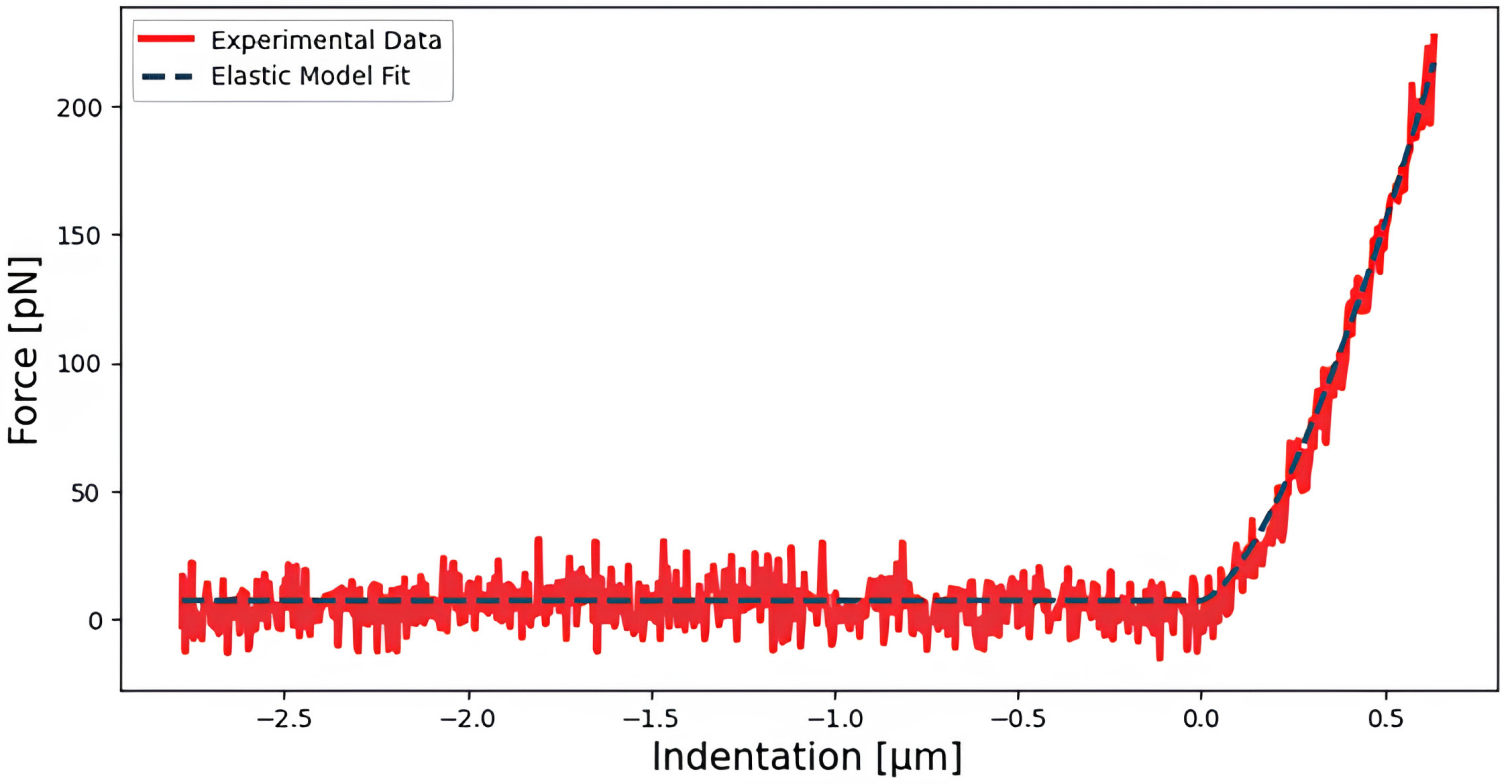
Approach segment of a force *vs* indentation curve acquired on a HeLa cell fitted to a Hertz paraboloidal elastic model, with an apparent Young’s modulus equal to 886 Pa.

***Determination of the Young’s modulus of living HeLa cells***. The Young’s modulus ( *E*) of 20 HeLa cells was measured, as shown in Figure 3, by fitting the paraboloidal Hertz’s contact model ( Table 1) to the approach segment of each force-distance curve of the dataset A. All three software packages were used for this analysis and the results were used for comparison. Other software packages openly available to extract the Young’s modulus, such as AtomicJ ( Hermanowicz *et al.*, 2014) or pyJibe ( Müller *et al.*, 2019), led to similar results.

**Table 1.** Implemented contact elastic relationships between force ( *F*) and indentation ( *δ*). *R* corresponds to the tip radius, *v* to the Poisson’s ratio, *E* to the Young’s modulus and *θ* to the semi-opening angle of the cone or the pyramid face.

Tip geometry	Formula	Reference
Paraboloidal	$\text{F}\left(\delta \right)\;=\;\frac{4\sqrt{R}}{3\left(1–v^{2}\right)}\;E\delta^{\frac{3}{2}}$	( Hertz, 1882)
Conical	$\text{F}\left(\delta \right)\;=\;\frac{2\tan\theta }{\pi \left(1–v^{2}\right)}\;E\delta^{2}$	( Love, 1939; Sneddon, 1965)
4-sided regular pyramid	$\text{F}\left(\delta \right)\;=\;\frac{\tan\theta }{\sqrt{2}\left(1–v^{2}\right)}\;E\delta^{2}$	( Barber & Billings, 1990; Bilodeau, 1992)

**Table 2.** Implemented bottom effect corrected elastic relationships between force ( *F*) and indentation ( *δ*). *R* corresponds to the tip radius, *v* to the Poisson’s ratio, *E* to the Young’s modulus, *h* to the sample thickness and *θ* to the semi-opening angle of the cone or the pyramid face.

Tip geometry	Formula	Reference
Paraboloidal	$$\begin{array}{l} F\left(\delta \right)\;=\;F_{0}\left(\delta \right)[\frac{1}{h^{0}}\;+\;\frac{1.133\sqrt{\delta R}}{h}\;+\;\frac{1.497\delta R}{h^{2}}\\ \;\;+\;\frac{1.469\delta R\sqrt{\delta R}}{h^{3}}\;+\;\frac{0.755(\delta^{2}R^{2})}{h^{4}}]\\ \;F_{0}\left(\delta \right)\;=\;\frac{4\sqrt{R}}{3\left(1–v^{2}\right)}\;E\delta^{\frac{3}{2}} \end{array}$$	( Garcia & Garcia, 2018)
Conical	$$\begin{array}{l} F\left(\delta \right)\;=\;F_{0}\left(\delta \right)[\frac{1}{h^{0}}\;+\;\frac{0.721\delta \tan\theta }{h}\;+\;\frac{0.650\delta^{2}{\left(\tan\theta \right)}^{2}}{h^{2}}\\ \;+\;\frac{0.491\delta^{3}{\left(\tan\theta \right)}^{3}}{h^{3}}\;+\;\frac{0.225\delta^{4}{\left(\tan\theta \right)}^{4}}{h^{4}}]\\ \;F_{0}\left(\delta \right)\;=\;\frac{2\tan\theta }{\pi \left(1–v^{2}\right)}\;E\delta^{2} \end{array}$$	
4-sided regular pyramid	$$\begin{array}{l} F\left(\delta \right)\;=\;F_{0}\left(\delta \right)[\frac{1}{h^{0}}\;+\;\frac{0.721\delta \tan\theta }{h}\;+\;\frac{0.650\delta^{2}{\left(\tan\theta \right)}^{2}}{h^{2}}\\ \;\;+\;\frac{0.491\delta^{3}{\left(\tan\theta \right)}^{3}}{h^{3}}\;+\;\frac{0.225\delta^{4}{\left(\tan\theta \right)}^{4}}{h^{4}}]\\ \;F_{0}\left(\delta \right)\;=\;\frac{2\tan\theta }{\sqrt{2}\left(1–v^{2}\right)}\;E\delta^{2} \end{array}$$	

**Table 3.** Implemented numerical viscoelastic relationships between force ( *F*) and indentation ( *δ*). *R* corresponds to the tip radius, *v* to the Poisson’s ratio, *E* to the elastic relaxation modulus, *t*_1_ to the integration auxiliary function and *θ* to the semi-opening angle of the cone or the pyramid face.

Tip geometry	Force curve segment	Equation	Reference
Paraboloidal	Approach	$F\left(t,\delta \left(t\right)\right)\;=\;\frac{4\sqrt{R}}{3\;(1–v^{2})}\;\int_{0}^{t}E\left(t–\xi \right)\frac{\partial \delta^{\frac{3}{2}}}{\partial \xi }\;d\xi$	( Efremov *et al.*, 2017)
	Retract	$F\left(t,\delta \left(t\right)\right)\;=\;\frac{4\sqrt{R}}{3\;(1–v^{2})}\;\int_{0}^{t_{1}\left(t\right)}E\left(t–\xi \right)\frac{\partial \delta^{\frac{3}{2}}}{\partial \xi }\;d\xi$	
Conical	Approach	$F\left(t,\delta \left(t\right)\right)\;=\;\frac{2\tan\theta }{\pi \;(1–v^{2})}\;\int_{0}^{t}E\left(t–\xi \right)\frac{\partial \delta^{2}}{\partial \xi }\;d\xi$	
	Retract	$F\left(t,\delta \left(t\right)\right)\;=\;\frac{2\tan\theta }{\pi \;(1–v^{2})}\;\int_{0}^{t_{1}\left(t\right)}E\left(t–\xi \right)\frac{\partial \delta^{2}}{\partial \xi }\;d\xi$	
4-sided regular pyramid	Approach	$F\left(t,\delta \left(t\right)\right)\;=\;\frac{\tan\theta }{\sqrt{2}\;(1–v^{2})}\;\int_{0}^{t}E\left(t–\xi \right)\frac{\partial \delta^{2}}{\partial \xi }\;d\xi$	
	Retract	$F\left(t,\delta \left(t\right)\right)=\frac{\tan\theta }{\sqrt{2}(1–v^{2})}\int_{0}^{t_{1}(t)}E\left(t–\xi \right)\frac{\partial \delta^{2}}{\partial \xi }d\xi$	

**Table 4.** Implemented viscoelastic analytical relationships between force ( *F*) and time ( *t*) assuming power law rheology ( *E*( *t*) = *E*_0_( *t*/ *t*_0_) ^–β^). *Γ* corresponds to the gamma function, *R* to the tip radius, *θ* to the semi-opening angle of the cone or the pyramid face, *v* to the Poisson’s ratio, *E*_0_ to the scaling factor or instantaneous elastic modulus, *β* to the power law exponent or fluidity, *t*_0_ to the scaling time, *t*_1_ to the integration auxiliary function, *v*_a_ to the approach velocity and *C~* to the tip geometry coefficients are defined in Table 5.

Tip geometry	Force curve segment	Formula	Reference
Paraboloidal	Approach	$\text{F}\left(t\right)\;=\;\frac{{v_{a}}^{\frac{3}{2}}}{C\sim }\;E_{0}\frac{{t_{0}}^{\beta }3\sqrt{\pi }\Gamma \left(1\;–\beta \right)}{4\Gamma \left(\frac{5}{2}–\beta \right)}\;t^{\frac{3}{2}–\beta }$	Based on ( Brückner *et al.*, 2017)
	Retract	$\text{F}\left(t\right)\;=\;\frac{1}{C\sim }\;E_{0}{v_{a}}^{\frac{3}{2}}{\left(\frac{t}{t_{0}}\right)}^{–\beta }\;t_{1}^{\frac{3}{2}}2F1\;\left(\frac{3}{2},\beta ;\frac{5}{2};\frac{t_{1}}{t}\right)$	
Conical / 4-sided regular pyramid	Approach	$\text{F}\left(t\right)\;=\;2\;\frac{{v_{a}}^{2}}{C\sim }\;E_{0}\frac{{t_{0}}^{\beta }\Gamma \left[2\right]\Gamma \left[1\;–\beta \right]}{\Gamma \left(3–\beta \right)}\;t^{2–\beta }$	
	Retract	$$\begin{array}{l} \text{F}\left(t\right)\;=\;2\;\frac{v_{r}^{2}E_{0}t_{0}^{\beta }}{C\sim \left(2–3\beta +\beta^{2}\right)}[t^{2–\beta }\\ \;+\;{\left(t–t_{1}\right)}^{1\;–\;\beta }\;\left(t_{1}+\left(1+\beta \right)t\right)] \end{array}$$	

**Table 5.** Geometry coefficients used in the implemented viscoelastic analytical models ( Table 4). *R* corresponds to the tip radius and *θ* to the semi-opening angle of the cone or the pyramid face.

Tip geometry	Coefficient
Paraboloid	$C\sim \;=\;3\;\left(1–v^{2}\right)/\left(4\sqrt{R}\right)$
Cone	$C\sim \;=\pi \;\left(1–v^{2}\right)/\left(2\tan\theta \right)$
4-sided regular pyramid	$C\sim \;=1.342\;\;\left(1–v^{2}\right)/\tan\theta$

**Table 6.** Values of the storage modulus ( *G′*), loss modulus ( *G″*) and loss tangent ( *η*) for purely elastic and viscous materials. *E* is the Young’s modulus, *v* is the Poisson’s ratio and *ω* is the angular frequency.

Purely elastic material	Purely viscous material
$G^{\prime }\left(\omega \right)\;=\;\frac{E}{2\left(1+v\right)}$	*G′* = 0
*G″*( *ω*) = 0	*G″*( *ω*) = μ
*η*( *ω*) = 0	*η*( *ω*)→∞

**Table 7.** Implemented frequency domain rheological models for the complex shear modulus ( G*) as a function of frequency ( *ω*). Where *F*( *ω*) and *δ*( *ω*) are the Fourier transform of the force and indentation, respectively, *δ*_0_ corresponds to the working indentation at which small amplitude oscillations are applied, *R* corresponds to the tip radius and *θ* to the semi-opening angle of the cone or the pyramid face.

Geometry	Function	Reference
Paraboloidal	$G^{*}\left(\omega \right)\;=\;\frac{1\;–v}{4{\left(R\delta_{0}\right)}^{\frac{1}{2}}}\;\frac{F\left(\omega \right)}{\delta \left(\omega \right)}$	( Mahaffy *et al.*, 2000; Rico *et al.*, 2005)
Pyramidal	$G^{*}\left(\omega \right)\;=\;\frac{1\;–v}{3\delta_{0}\tan\theta }\;\frac{F\left(\omega \right)}{\delta \left(\omega \right)}$	( Alcaraz *et al.*, 2003)

**Table 8.** Open-source Python libraries used by PyFMLab.

Library name	Description	References
Numpy	Numerical computation and N-dimensional arrays.	( Harris *et al.*, 2020)
Pandas	Data manipulation and DataFrame data structures.	( McKinney, 2010)
SciPy	Algorithms for scientific computing.	( Virtanen *et al.*, 2020)
Lmfit	Non-Linear least-squares minimization and curve-fitting.	( Newville *et al.*, 2023)
PyQT 5	Graphical user interface framework.	https://github.com/PyQt5
PyQTGraph	User interface graphics.	https://github.com/pyqtgraph
PyInstaller	Freezing PyFMLab code for distribution.	https://github.com/pyinstaller

**Figure 4.**
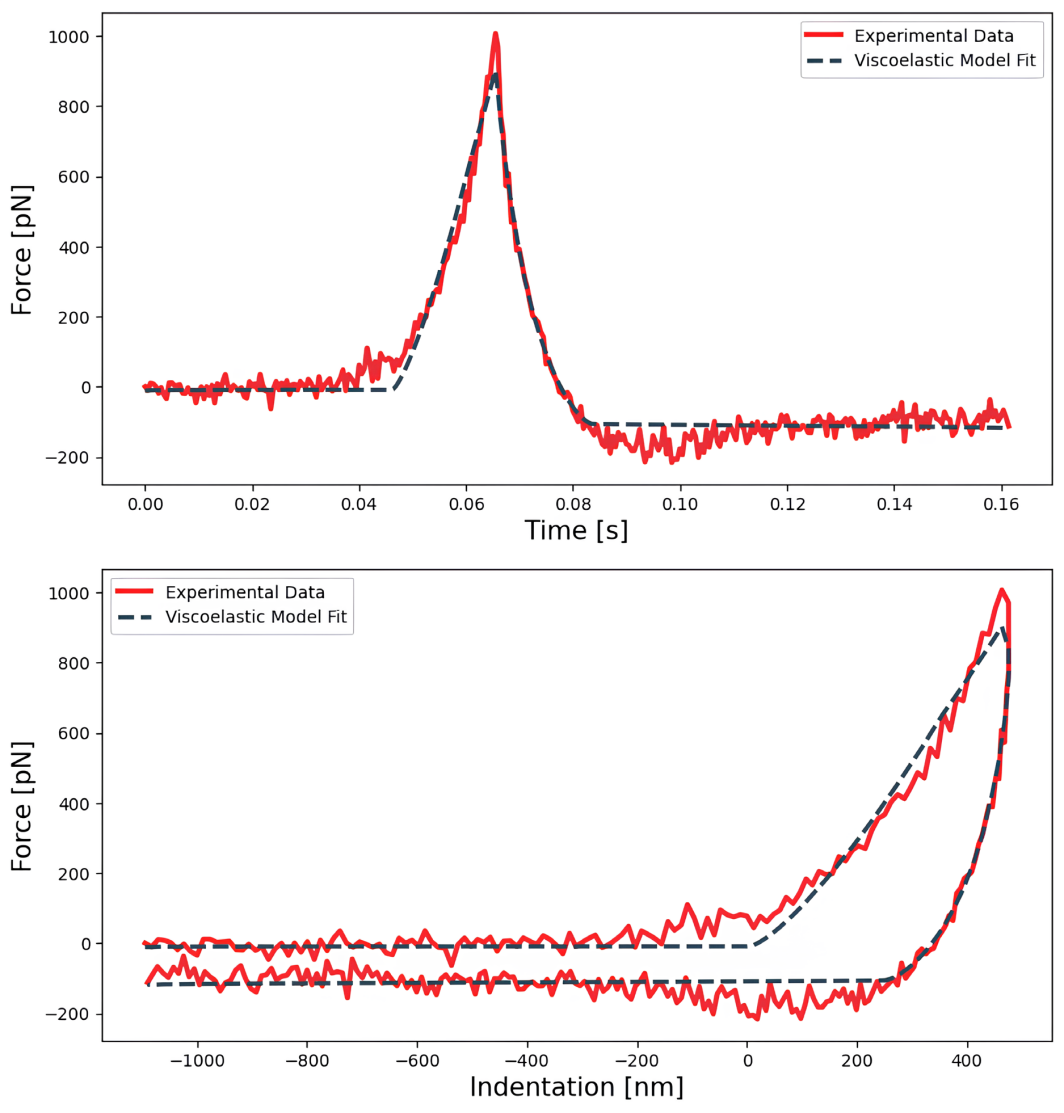
Force indentation curve acquired on a HeLa cell fitted to an analytic paraboloidal viscoelastic model, with an instantaneous elastic modulus ( *E*_0_) and fluidity exponent of 334 Pa and 0.17 respectively. The fit is done on the force *vs* time curve (top) and can then be displayed on the force *vs* indentation curve (bottom), allowing a direct comparison with the elastic fit shown on Figure 3.

***Determination of the viscoelasticity of living HeLa cells.*** The same dataset A (simple force curves) was used to compute the scaling factor *E*_0_ and the power-law fluidity *β* exponent by fitting the analytical model developed by Brückner *et al.*, 2017 for a paraboloidal tip ( Table 4), as shown in Figure 4.

**Figure 5.**
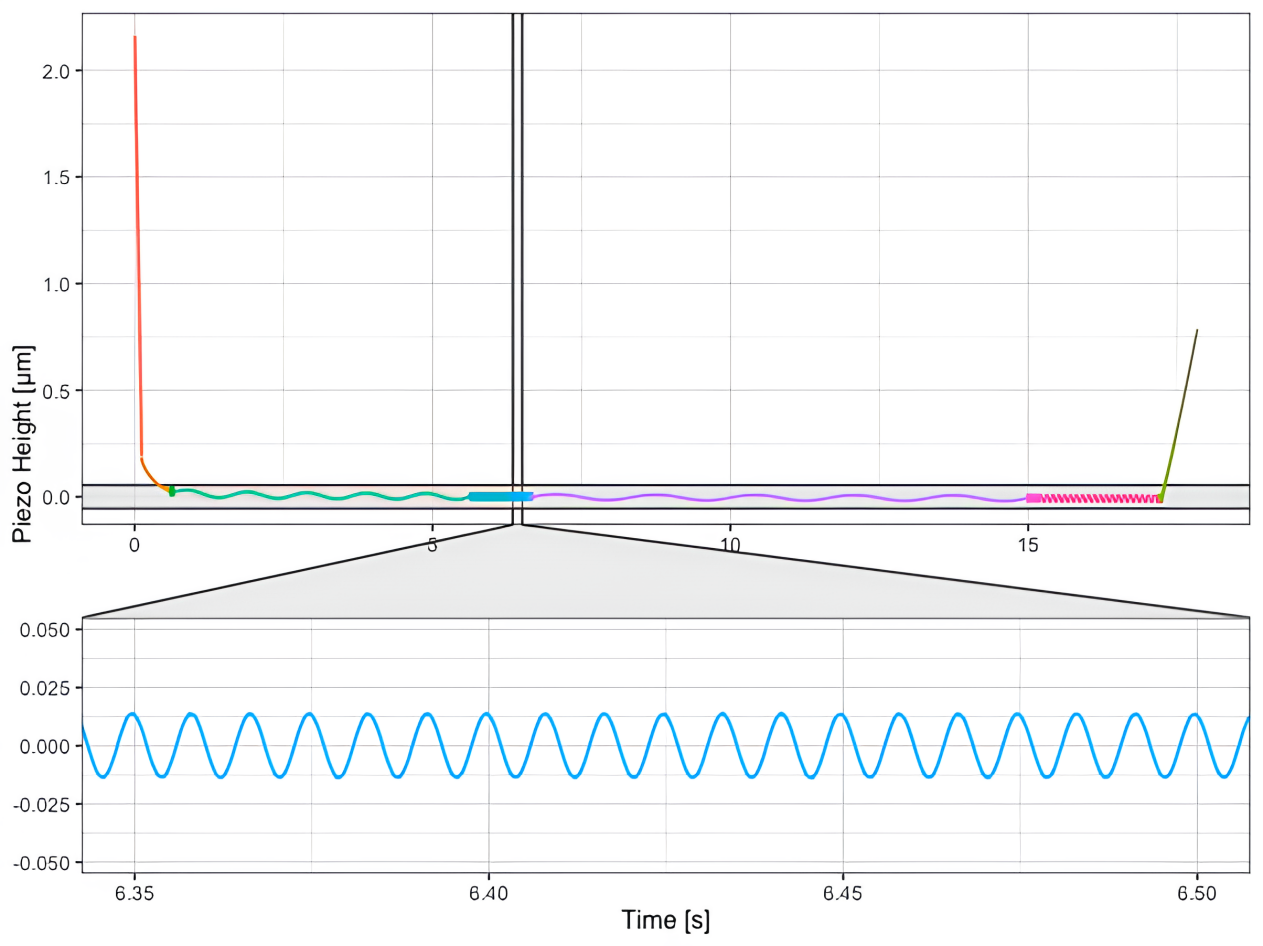
Microrheology data acquired on a HeLa cell. The cantilever is brought to contact with the sample up to reach the working indentation (red portion of the curve) and then oscillated at different frequencies (depicted in different colors, green, blue, violet, rose portions of the curve) before to be retracted from the sample (green portion of the curve). The inset shows the sinusoidal oscillations of the cantilever at a single frequency.

Only the MATLAB routines and PyFMLab were used for this analysis. The standard error for *E*_0_ values obtained from the two packages were calculated using bootstrapping, by computing a two-sided bootstrap confidence interval of the *E*_0_ median (Scipy).

***Microrheological measurements on HeLa cells.*** For this analysis, the dataset B (oscillatory measurements) acquired on 20 HeLa cells was used to compute the power-law parameters (scaling factor *G*_0_ and fluidity exponent *β*). To correct for the effect of the viscous drag and Z-piezo artefacts, the phase lag and amplitude quotient of the Z-piezo, together with the drag force at contact, were determined using PyFMLab ( Figure 8).

**Figure 6.**
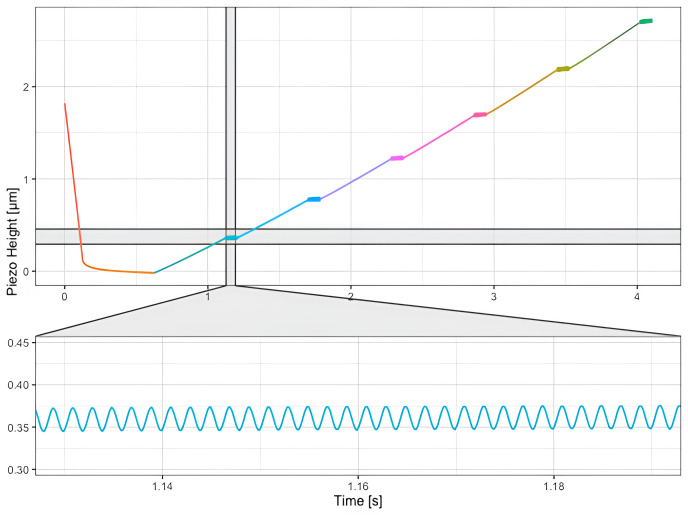
Data acquired on HeLa cells, using a SAA-SPH-5UM AFM ( Figure 7) paraboloidal probe (Bruker, Santa Barbara, CA, USA), to determine the viscous drag. The cantilever is brought into contact with the sample (h = 0 μm, orange portion of the curve) and retracted 6 times (segments depicted in 6 different colors) until the maximum tip-sample distance of 2.75 μm is reached. After every retract, the cantilever is oscillated at the same frequency. This modulation step is shown in the inset.

**Figure 7.**
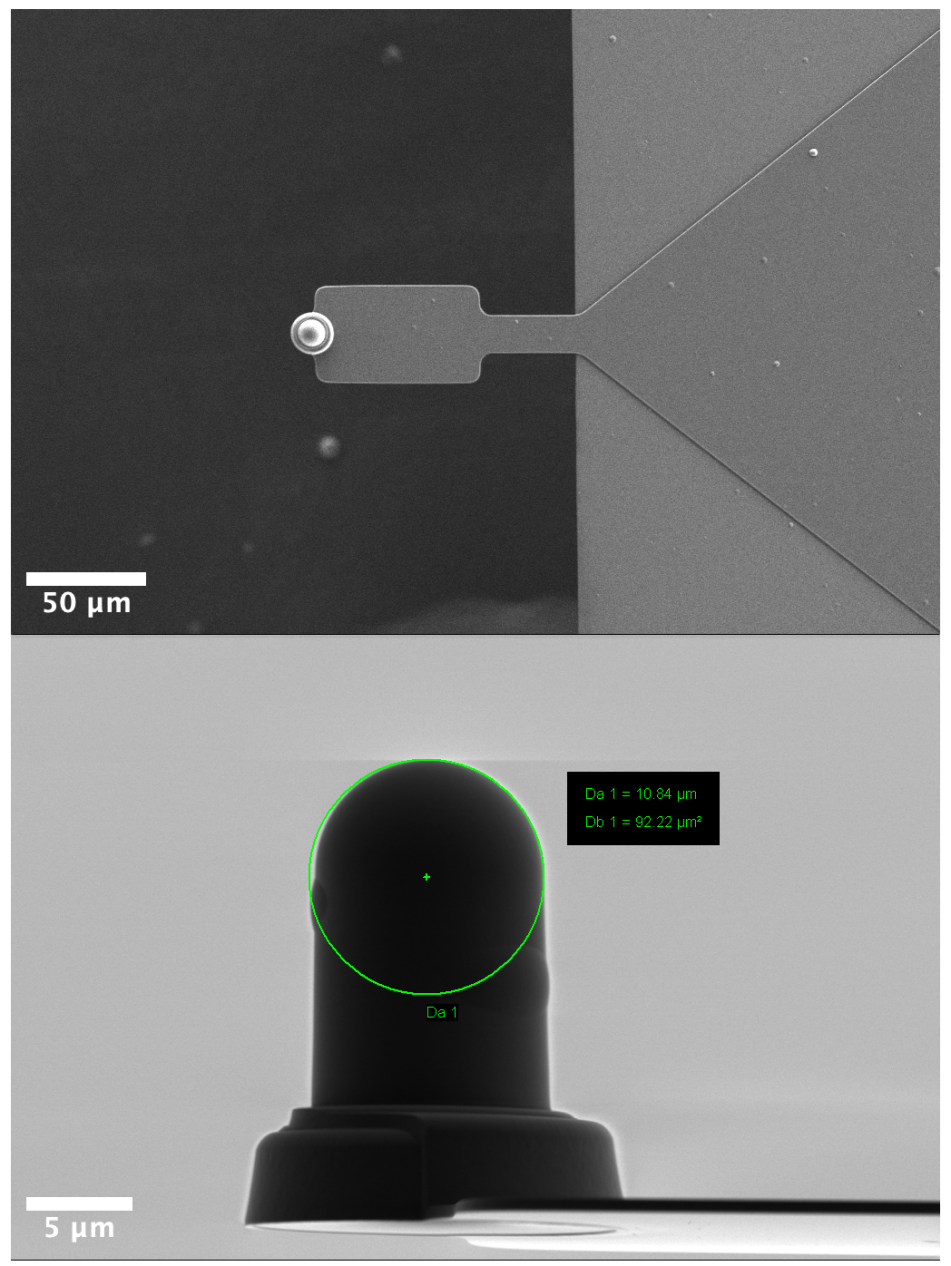
Scanning electron microscopy images of the SAA-SPH-5UM AFM probe (Bruker, Santa Barbara, CA, USA). Top view (top) Side view (bottom).

**Figure 8.**
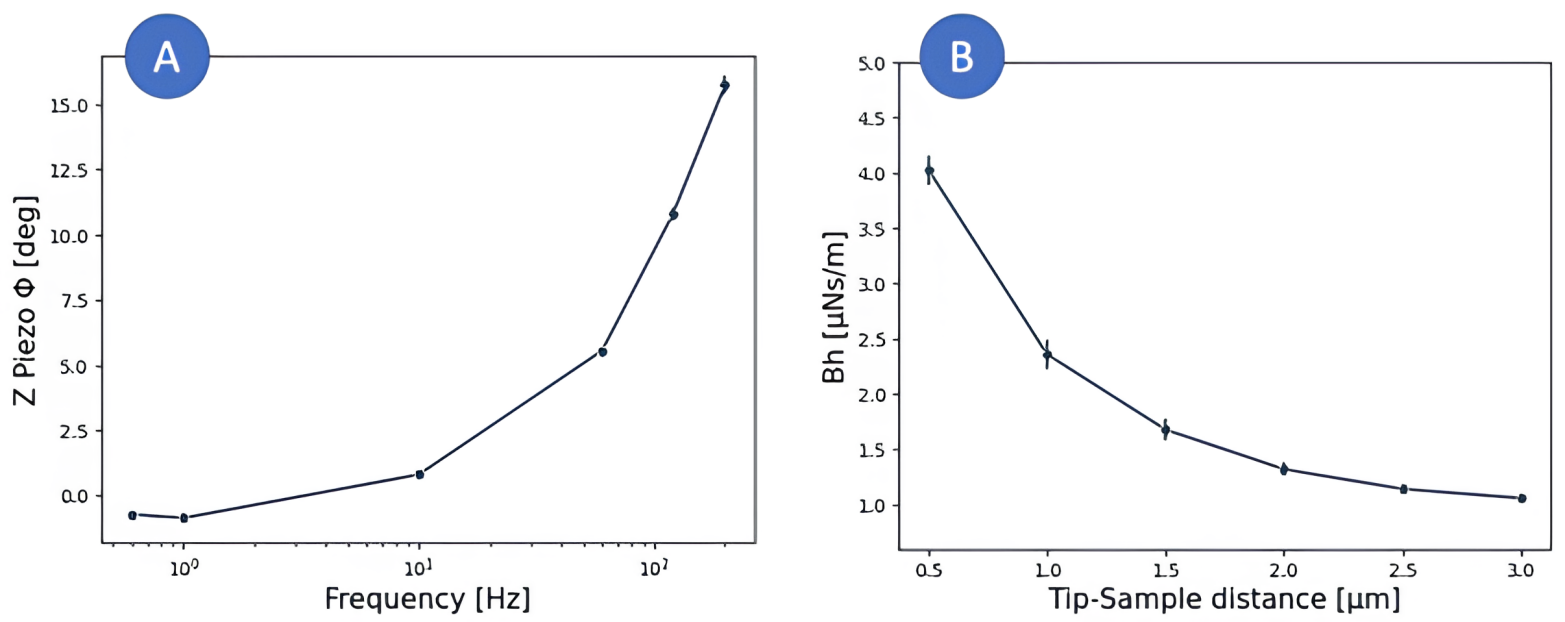
**A**) Z-piezo phase lag measured on a NanoWizard III for each frequency. **B**) Drag force measured at different cantilever-sample separations while applying 500 Hz oscillations. The data shown on each plot corresponds to the mean ± SE of three 4x4 force curves maps.

The geometric mean of the G*( *f*) for each cell was then fitted to a double power law:


$$\begin{array}{l} G^{\prime }\left(f\right)=A\cos\left(\pi \alpha /2\right)f^{\alpha }+B\cos\left(\pi \beta /2\right)f^{\beta }\\ G^{\prime \prime }\left(f\right)=A\sin\left(\pi \alpha /2\right)f^{\alpha }+B\sin\left(\pi \beta /2\right)f^{\beta } \end{array}$$

**Figure 9.**
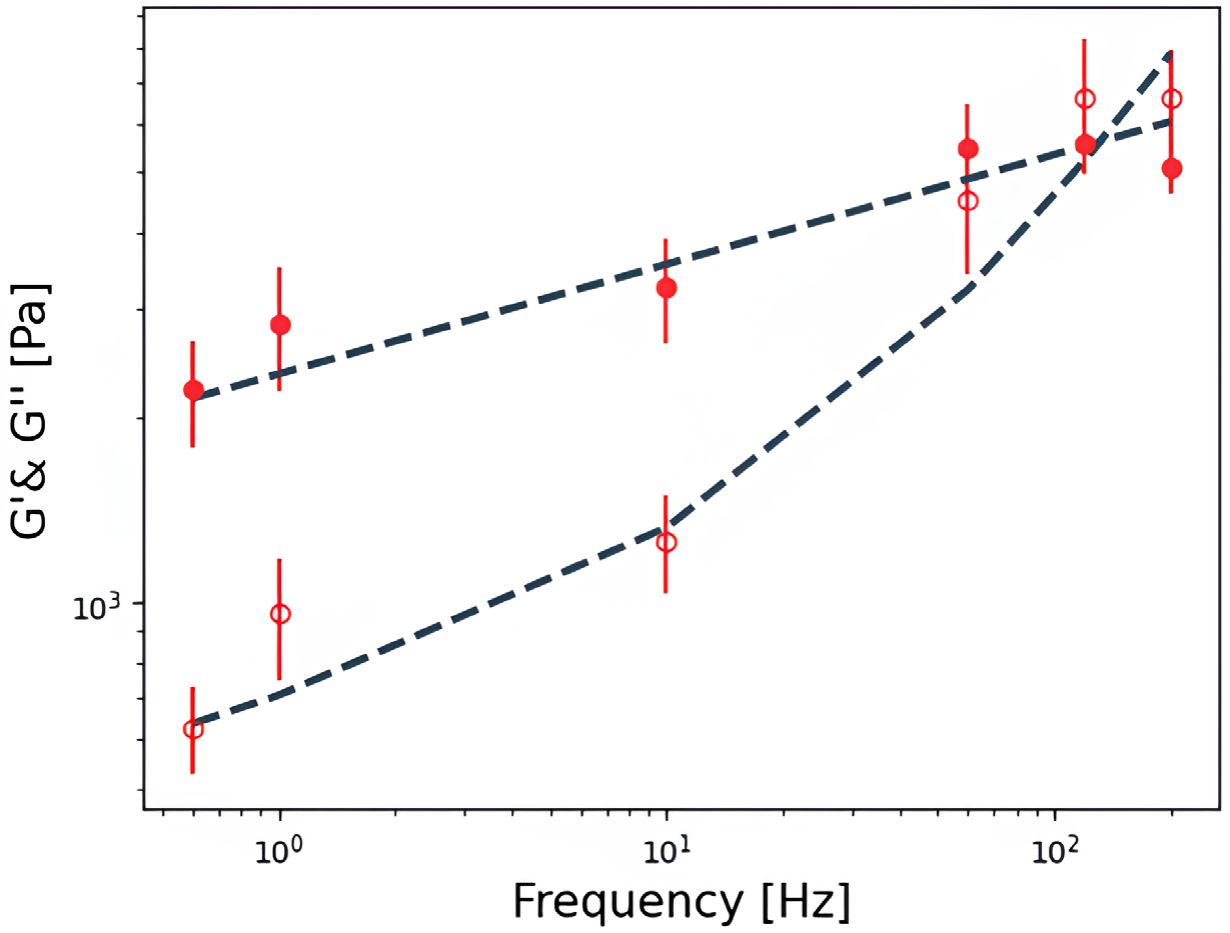
Experimental *G*'( *f*) (filled symbols) and *G*''( *f*) (hollow symbols) data of a cell fitted to a double power law. Each point shows the geometric mean ± SE of three 4x4 force curves map. The dashed line is a fit to the double power law with fitted parameters A, B, α and, *β* equal to 2663 Pa, 38 Pa, 0.17 and 0.99, respectively.

By least squares minimization using the Lmfit ( Newville *et al.*, 2023) Python 3 library, leaving the four parameters A, B, α and, *β* free ( Figure 9). Microrheology measurements were only processed using PyFMLab.

### Scanning electron microscopy

AFM probes were mounted on stub with a carbon double-side tape then observed with a Zeiss Merlin Compact FESEM (Zeiss, France) working at 1 kV.

### Results

**Figure 10.**
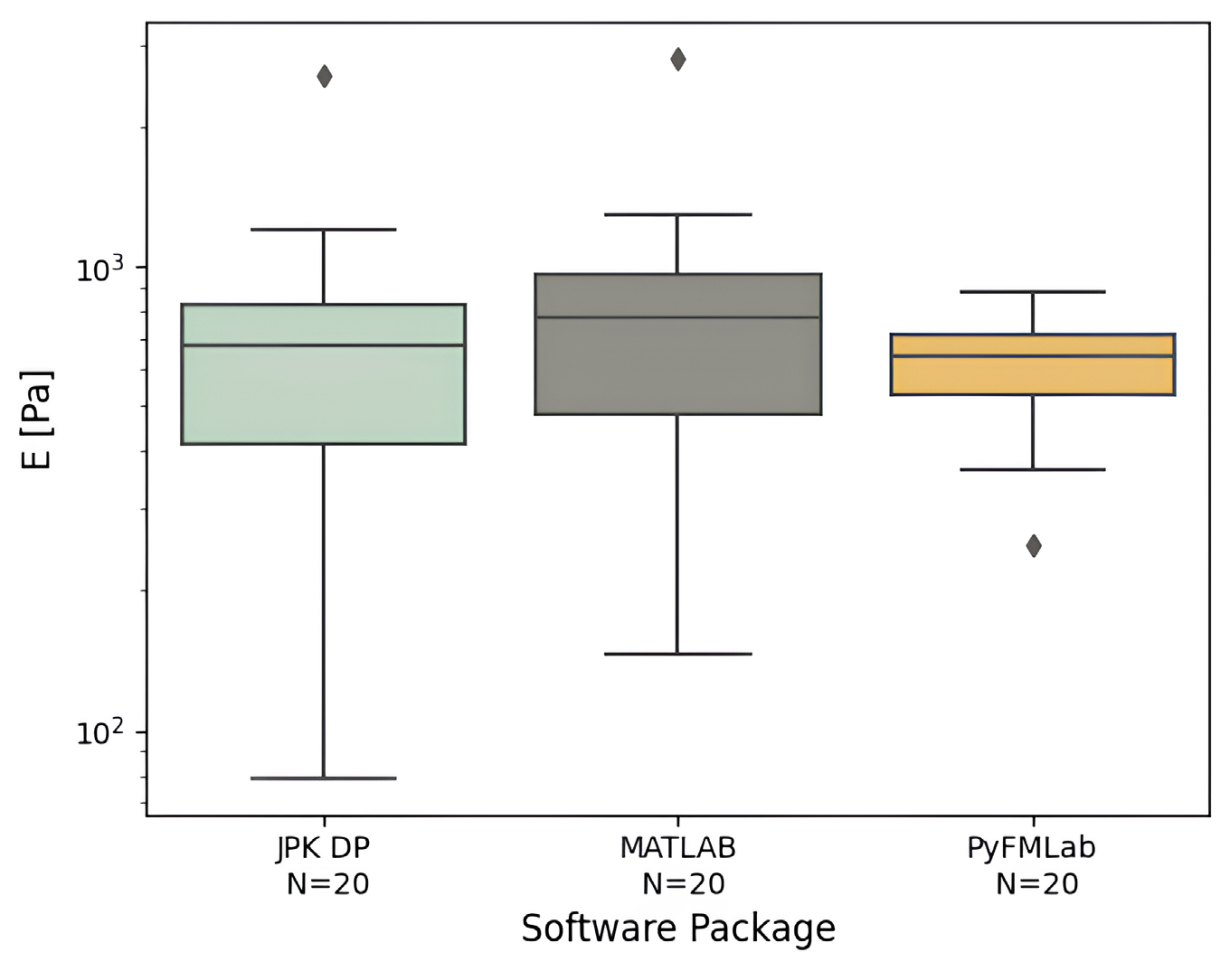
Median apparent Young’s modulus ( *E*) of individual HeLa cells (N=20 cells, with 16 measurements per cell) measured by different software packages. Centre line in the box plots represents the median value, top and bottom limits of the box represent the first and third quartiles (25% and 75%) of the data. Whiskers represent minimum and maximum values; diamonds represent the outliers (points outside 1.5 times the interquartile range).

***Comparison between apparent Young’s modulus values.*** To validate the elastic model fit of PyFMLab, a dataset comprising measurements of 20 HeLa cells was analyzed using three different software packages ( Figure 10). From the analysis the following results were obtained: JPK DP *E*_0_ = 676 Pa ± 93 Pa (median ± SE), MATLAB routines *E*_0_ = 776 Pa ± 110 Pa and PyFMLab *E*_0_ = 641 Pa ± 32 Pa. Where SE refers to the standard error, computed through bootstrapping (10000 resamples). All values are in agreement with each other and vary in a range of 4–17%, with the MATLAB routines and PyFMLab showing the greatest difference (135 Pa). PyFMLab allows more robust determination of the contact point thanks to the RoV method ( Gavara, 2016). This ensures less dispersion of the *E*_0_ results, whereas the fit errors were wider for the previous methods.

***Comparisons between power-law fluidity exponent values obtained.*** The power-law fluidity exponent was measured on HeLa cells through both a time-domain and a frequency-domain method.

**Figure 11.**
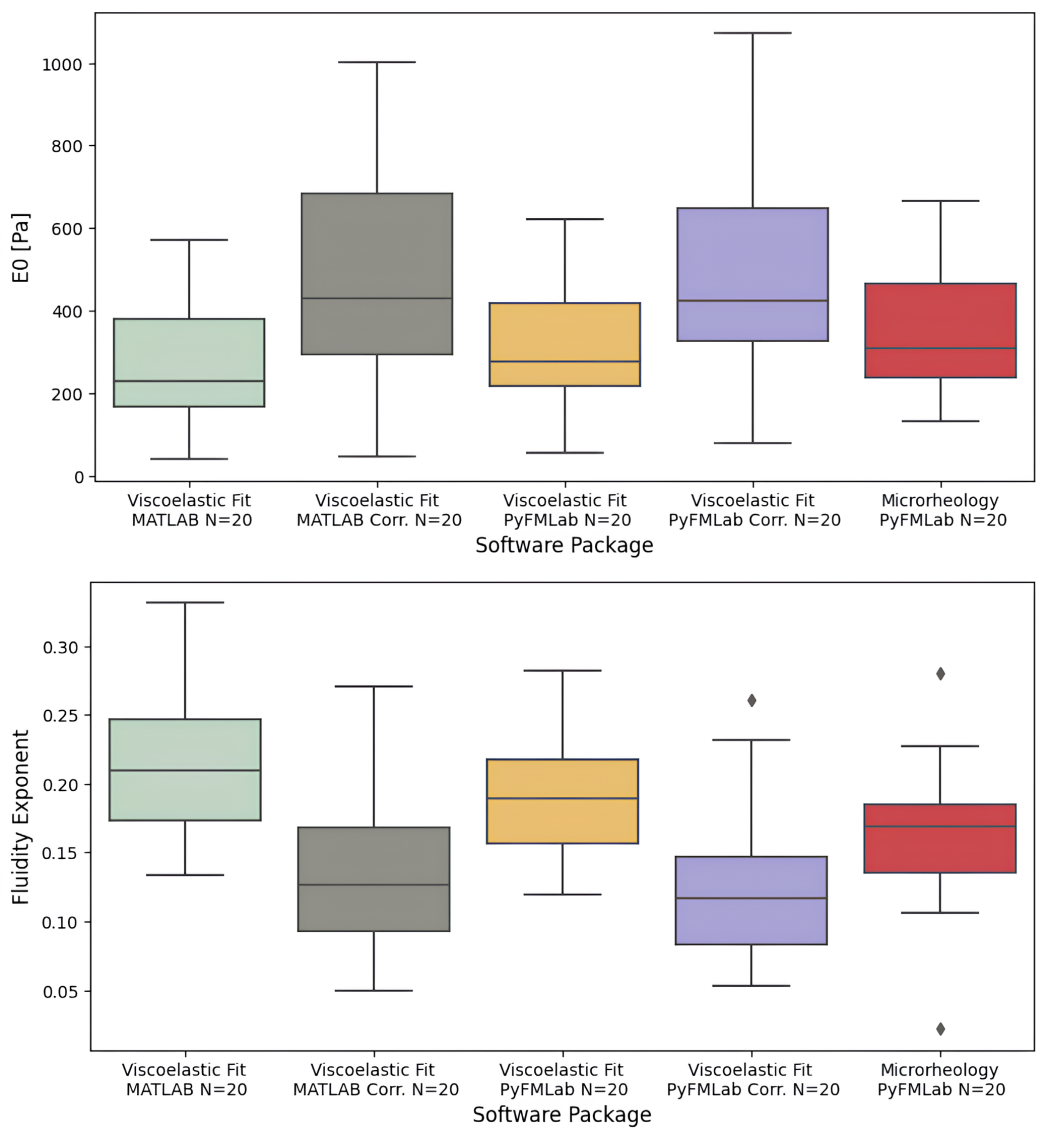
Instantaneous elastic modulus ( *E*_0_) (Top) and Power-law fluidity exponent (Bottom) of individual HeLa cells (N=20 cells, 16 measurements per cell) measured by different software packages. The values obtained from fitting the viscoelastic model represent the median *E*_0_ and *α* with and without correcting for the viscous drag assuming a viscous drag of 0.003 pN nm ^-1^ s. The values reported obtained from the microrheology measurements represent the fluidity exponent obtained by fitting the double power law model to the geometric mean for each cell. *G* ( *f*) was corrected for the z-piezo phase lag, signal amplitude and hydrodynamic drag ( *b*(0) = 0.005 pN s nm ^-1^). Centre line in the box plots represents the median value, top and bottom limits of the box represent Q1 and Q3 of the data. Whiskers represent minimum and maximum value; diamonds represent the outliers (points outside 1.5 times the interquartile range).

For the time-domain method, the dataset A was used to fit the viscoelastic model developed by Brückner *et al.* ( Brückner *et al.*, 2017) to the full approach-retract cycle (as shown on Figure 4). The model was fitted using both the MATLAB routines and PyFMLab ( Figure 11). As the measurements were performed in liquid, the data was corrected assuming a viscous drag factor of 0.003 pN nm ^-1^ s. The following values were obtained from the analysis: Viscoelastic Fit MATLAB *α* = 0.21 ± 0.018 (median ± SE), Viscoelastic Fit MATLAB Corrected *α* = 0.13 ± 0.018, Viscoelastic Fit PyFMLab *α* = 0.18 ± 0.014, Viscoelastic Fit PyFMLab Corrected *α* = 0.12 ± 0.013. Where SE refers to the standard error, computed through bootstrapping (10000 resamples). As expected, the values corrected for the viscous drag were lower for fluidity exponent and higher for *E*_0_ than the non-corrected ones. Nevertheless, the values obtained from both routines are in agreement and in the range of *α* values that other authors have reported for cells ( Brückner *et al.*, 2017; Efremov *et al.*, 2017; Flormann *et al.*, 2021).

For the frequency-domain method, the dataset B, acquired on 20 other HeLa cells, was used. During the analysis, the measurements were corrected for the z-piezo phase lag, amplitude quotient and hydrodynamic drag ( *b*(0) = 0.005 pN s nm ^-1^). A *α* of 0.17 ± 0.013 (median ± SE) was measured. These values are within the range of values that other authors have reported for HeLa cells ( Flormann *et al.*, 2021; Rother *et al.*, 2014) and are similar to the values obtained from the viscoelastic fits. This trend was previously observed by other authors ( Efremov *et al.*, 2017).

## Discussion

In this work, an open-source software package has been developed allowing us to measure the elastic and viscoelastic properties of soft samples from FDCs and microrheological measurements. The modularity of PyFMLab provides a user the possibility to use the package as a turn-key solution or to implement their custom features.

To validate the implemented algorithms, experimental data acquired on living HeLa cells was processed using three different software packages: commercial JPK DP, custom MATLAB routines and PyFMLab. The median apparent Young’s modulus ( *E*) of 20 cells was obtained using all three software packages. All results obtained are in agreement with each other and differ in a range of 4 – 17%.

The viscoelastic properties of HeLa cells were characterized by both fitting a viscoelastic model to the approach-retract cycle (time-domain method) and by performing microrheological measurements (frequency-domain method). The viscoelastic model developed by Brücker *et al.* ( Brückner *et al.*, 2017) was fitted to the approach-retract cycle of the FDCs to determine *E*_0_ and the fluidity exponent using both the MATLAB routines and PyFMLab on the same dataset. Microrheology data was processed using PyFMLab. Values obtained for *E*_0_ and the power-law fluidity exponent were in agreement both between software packages and methodologies. This opens the way to characterizing the rheological behavior of samples directly from datasets consisting of regular approach-retract force curves, for example, for high resolution mechanical maps. Thus, speeding data acquisition and analysis when compared to DMA experiments.

Although version 1.0 of PyFMLab includes the most widely used models and functionalities needed for mechanically characterizing biological samples, further improvements could be performed in the determination of the point of contact in the microrheology routine and other models to describe G* could be implemented. A separation dependent viscous drag correction may be also added to the FDC based approach. Furthermore, implementation of thickness determination from AFM mechanical maps will allow accurate correction of the bottom effect across the cell surface.

In conclusion, PyFMLab allows determination of viscoelasticity of biological samples from both force-distance curves and microrheology measurements. We expect that PyFMLab will become a standard in the field, given its versatility, open-source nature, modularity and robustness.

## Ethics and consent

Ethical approval and consent were not required.

## Data Availability

Zenodo: AFM Dataset on HeLa cells https://doi.org/10.5281/zenodo.8342121 ( López-Alonso, 2023a). This project contains the following underlying data: Force Settings (HeLaDataset_20221029/ForceSettings) Approach-Retract curves acquired on glass (HeLaDataset_20221029/Glass) Oscillatory measurements acquired on HeLa cells (HeLaDataset_20221029/Microrheo) Oscillatory measurements acquired on glass for performing the piezo characterization (HeLaDataset_20221029/PiezoChar) Approach-Retract curves acquired on HeLa cells (HeLaDataset_20221029/SFC) Therma data acquired for non-contact calibration (HeLaDataset_20221029/Thermal) Oscillatory measurements acquired on the medium over HeLa cells to compute the viscous drag coefficient (HeLaDataset_20221029/VDrag) Results obtained from JPKDP v.7.1.23 (JPKDP_Results) Results obtained from MATLAB routines (MATLAB_Results) Results obtained from PyFMLab (PyFMLab_Results) Data are available under the terms of the Creative Commons Attribution 4.0 International license (CC-BY 4.0).

## Software availability

### PyFMReader

Archived source code at the time of publication: https://doi.org/10.5281/zenodo.8342163 ( López-Alonso, 2023b)

Source code available from: https://github.com/jlopezalo/PyFMReader

License: MIT

***PyFMRheo***

Archived source code at the time of publication: https://doi.org/10.5281/zenodo.8342163 ( López-Alonso, 2023b)

Source code available from: https://github.com/jlopezalo/PyFMRheo

License: MIT

### PyFMGUI

Archived source code at the time of publication: https://doi.org/10.5281/zenodo.8342163 ( López-Alonso, 2023b)

Source code available from: https://github.com/jlopezalo/PyFMGUI

License: MIT
