# Supplementary material for: PyFMLab: Open-source software for atomic force microscopy microrheology data analysis
Source: Open Res Eur. 2024 Jul 24;3:187. Originally published 2023 Oct 30. [Version 2] doi: 10.12688/openreseurope.16550.2 (PMC11308986; doi:10.12688/openreseurope.16550.2)
Supplement: Peer review history

# Reviewer report: Reviewer response for version 2

**DOI:** 10.21956/openreseurope.19086.r42420

**Published:** 2024-08-05

**Authors:** Stylianos Vasileios Kontomaris

**Affiliations:** 1 Metropolitan College, Athens, Greece

I congratulate the authors on this very useful paper for the entire AFM community. I have no further comments.

Is the rationale for developing the new software tool clearly explained?

Yes

Is the description of the software tool technically sound?

Yes

Are sufficient details of the code, methods and analysis (if applicable) provided to allow replication of the software development and its use by others?

Yes

Is sufficient information provided to allow interpretation of the expected output datasets and any results generated using the tool?

Yes

Reviewer Expertise:

AFM mechanical characterization, mathematical modelling

I confirm that I have read this submission and believe that I have an appropriate level of expertise to confirm that it is of an acceptable scientific standard.

# Reviewer report: Reviewer response for version 2

**DOI:** 10.21956/openreseurope.19086.r42421

**Published:** 2024-07-24

**Authors:** Mi Li

**Affiliations:** 1 Shenyang Institute of Automation Chinese Academy of Sciences, Beijing, China

My concerns have been well addressed and I have no further comments.

Is the rationale for developing the new software tool clearly explained?

Yes

Is the description of the software tool technically sound?

Yes

Are sufficient details of the code, methods and analysis (if applicable) provided to allow replication of the software development and its use by others?

Partly

Is sufficient information provided to allow interpretation of the expected output datasets and any results generated using the tool?

Yes

Reviewer Expertise:

atomic force microscopy, mechanobiology

I confirm that I have read this submission and believe that I have an appropriate level of expertise to confirm that it is of an acceptable scientific standard.

# Reviewer report: Reviewer response for version 1

**DOI:** 10.21956/openreseurope.17865.r36122

**Published:** 2023-12-08

**Authors:** Stylianos Vasileios Kontomaris

**Affiliations:** 1 Metropolitan College, Athens, Greece

This is a very important work in the field of AFM data processing regarding biological samples. As correctly indicated by the authors, there is currently no standardized method or software that can efficiently process all the data obtained from AFM. For example, powerful software packages (e.g. the AtomicJ software provide accurate results but do not provide all the information mentioned here, i.e. do not allow the processing of microrheology data).This is a significant drawback in AFM research, as many researchers are compelled to build home-made algorithms, which is a time-consuming procedure. In many cases, these algorithms lack accuracy. Therefore, this work could represent a significant step forward in streamlining data processing for AFM experiments.

 I have some questions/suggestions provided below:

 1) In Table 1, there are the standard elastic models for paraboloid, conical and 4 sided regular pyramids. The Hertz equation for parabolic indenters can also be applied to spherical indenters for small indentation depths (e.g., if the indentation depth h is h < R/10, where R is the indenter’s radius). However, in many cases, spherical indenters with a radius in the range of a few micrometers are used for data processing. In this case, for large indentation depths, the force-indentation data do not follow the first equation in Table 1. The same restriction also applies to sphero-conical indenters. Therefore, in my opinion, it should be emphasized in the article that these cases will be included in future versions of the software.

 2) I have been using AtomicJ software for several years, and it is a powerful tool for acquiring Young’s modulus. The authors clearly mentioned that both AtomicJ and the new software (PyFMLab) led to similar results. In my opinion, this is important. However, I believe that the percentage difference in Young’s modulus values between AtomicJ and PyFMLab should be added for comparison. It is significant to determine if the percentage difference between these two is 4%, 17%, or something similar.

 3) In the AFM measurements section, the authors explain that, for measuring the mechanical properties of cells, maps of force curves were acquired on the nucleus area of 20 cells. These maps had a size of 30 µm x 30 µm and a resolution of 4 × 4 pixels. I wonder why didn’t they use a standard map with 16 x 16 pixels (or even a map with 64 x 64 pixels) to provide a more ‘detailed’ mechanical characterization? If there is a specific reason for this choice, it should be clearly mentioned within the manuscript.

 4) The 4-17% difference between different software is explained based on the accuracy of the contact point determination. It is widely known that if we 'miss' the correct contact point, we can end up with a significant error, even around 100%. However, my question is whether this is the only source of these differences. If there are also other sources, they should be mentioned and discussed.

 5) In addition, the authors used HeLa cells. I wonder why they did not first validate the accuracy of their software using an approximately homogeneous and isotropic material, such as an agarose gel. The reason for this question is that I wonder if, in this case, the percentage difference between different software is minimized.

 6) Another point to mention is Figure 3. In this case the Hertz model fits almost perfectly the data (it seems that the R-squared coefficient is close to 1 in this case). My question is if all the tested data ‘perfectly followed’ a model of contact mechanics. As we know there are cases of force indentation curves in which the data only approximately follow the relative model. Therefore, what is the average R-squared coefficient in the tested data? Additionally, do you believe that in cases with low R-squared coefficients, the differences when using different algorithms will be affected or not?

Is the rationale for developing the new software tool clearly explained?

Yes

Is the description of the software tool technically sound?

Yes

Are sufficient details of the code, methods and analysis (if applicable) provided to allow replication of the software development and its use by others?

Yes

Is sufficient information provided to allow interpretation of the expected output datasets and any results generated using the tool?

Yes

Reviewer Expertise:

AFM mechanical characterization, mathematical modelling

I confirm that I have read this submission and believe that I have an appropriate level of expertise to confirm that it is of an acceptable scientific standard, however I have significant reservations, as outlined above.

# Reviewer report: Reviewer response for version 1

**DOI:** 10.21956/openreseurope.17865.r36129

**Published:** 2023-11-27

**Authors:** Mi Li

**Affiliations:** 1 Shenyang Institute of Automation Chinese Academy of Sciences, Beijing, China

This paper presents an open-source software named PyFMLab for the analysis of experimental data obtained during atomic force microscopy (AFM) mechanical assay. Various mechanical properties can be obtained by the software, including elasticity, viscoelasticity, and frequency-dependent microrheological mechanics. AFM has now become an important and standard tool for characterizing the mechanical properties of living biological specimens, but so far, nanomechanical studies with the use of AFM still mainly focus on characterization of single mechanical property such as elasticity. Cellular mechanical properties are highly complex, and cells possess multiple mechanical behaviors in addition to elasticity. Therefore, the software presented here which can analyze various mechanical properties of cells will benefit the field of AFM-based mechanobiology. I have several comments for the paper in its current form:

 1. The force curves in Fig. 3 and Fig. 4 are ideal force curves since the cantilever deflection keeps nearly zero before in contact with the cell, and it is easy to visually determine the contact point. In many cases, the obtained force curves during AFM mechanical experiments are not ideal, and baseline correction is often needed before the analysis of force curves. I think adding a baseline correction to the software will facilitate the analysis of experimental data.

 2. Table 2 shows the most commonly used elastic models (Hertz-Sneddon) for extracting the Young’s modulus of the specimen from force curves. Adding more theoretical models, such as JPK and DMT, which are also used in the field in case of the existence of adhesion between AFM tip and specimen, into the software will benefit comprehensively analyzing the force curves.

 3. Details of AFM-based oscillatory microrheology experiments are lacking, such as instrumental requirement, data collection, and data analysis.

 4. Besides Young’s modulus, the stiffness (the unit is N/m) calculated from the force curves is also used to characterize the rigidity/softness of specimen, and thus adding a function of extracting specimen stiffness from the force curves will be meaningful.

Is the rationale for developing the new software tool clearly explained?

Yes

Is the description of the software tool technically sound?

Yes

Are sufficient details of the code, methods and analysis (if applicable) provided to allow replication of the software development and its use by others?

Partly

Is sufficient information provided to allow interpretation of the expected output datasets and any results generated using the tool?

Yes

Reviewer Expertise:

atomic force microscopy, mechanobiology

I confirm that I have read this submission and believe that I have an appropriate level of expertise to confirm that it is of an acceptable scientific standard, however I have significant reservations, as outlined above.
